# Comparative analysis of text readability and writing styles in AI-generated vs. Human-written academic abstracts

**DOI:** 10.1371/journal.pone.0343163

**Published:** 2026-04-08

**Authors:** Yumei Zou, Florence Kuek, Kwan Hoong Ng, Xiaoli Cheng

**Affiliations:** 1 School of Foreign Languages, Jiangxi Agricultural University, Jiangxi, China; 2 Faculty of Arts and Social Sciences, Universiti Malaya, Kuala Lumpur, Malaysia; 3 Department of Biomedical Imaging, Faculty of Medicine, Universiti Malaya, Kuala Lumpur, Malaysia; 4 Faculty of Medicine and Health Sciences, UCSI University, Port Dickson, Negeri Sembilan, Malaysia,; 5 Nanchang University Gongqing College, Jiangxi, China; National University of Malaysia Faculty of Education: Universiti Kebangsaan Malaysia Fakulti Pendidikan, MALAYSIA

## Abstract

Research article abstracts are vital in scientific publications for readers to assess a study’s significance. The increasing use of AI tools, such as Kimi, ChatGPT and DeepSeek, to generate abstracts raises concerns about their readability and writing styles compared to human-written ones. The study aims to compare the differences in text readability and writing styles between human-written against AI-generated abstracts. A total of 150 abstracts of high-impact journal articles in the field of linguistics and computer science, 75 from each discipline, and another 150 AI-generated abstracts from the same corpus of articles served as the source texts for analysis. The Readability Scoring System, a computational tool, yielded readability and writing style metrics, while expert evaluation was performed to assess the quality of AI-generated academic abstracts. The quantitative data generated were analysed using SPSS 27 with non-parametric statistical methods. Key findings revealed: (1) AI-generated abstracts exhibited significantly lower readability across eight metrics, indicating greater complexity and lower readability; (2) Discipline-specific analysis showed five differing metrics in linguistics and eight in computer science; (3) Interdisciplinary comparisons revealed non-significant differences across nine readability metrics, highlighting AI’s potential to mimic natural writing. However, it still faces challenges in generating lexically diverse content. These results underscored the current limitations of AI in generating readable and human-like abstracts, especially in technical fields.

## 1. Introduction

The integration of Artificial Intelligence (AI) in academic writing has spurred increasing research evaluating AI tools’ performance [1–3]. Such evaluations are crucial for informing researchers on the use of large language models (LLMs) in academic contexts [4–6]. Previous studies indicate AI’s transformative impact on academic writing and research across disciplines [1–3]. Tools like ChatGPT and Kimi can generate coherent, contextually relevant texts in diverse fields [7], enhancing productivity [1,8] and at times, mimicking human writing convincingly [9,10].

However, key questions remain regarding the readability and writing styles of AI-generated text. While AI may produce structured and grammatically sound content, its ability to capture the nuanced, discipline-specific knowledge of high-quality academic writing is unclear. Furthermore, comprehensive comparative analyses of AI-generated and human-written abstracts across different academic disciplines are lacking, hindering our understanding of AI’s broader impact on scholarly communication.

To address these gaps, this study evaluated the effectiveness of AI tools in generating research article abstracts by comparing AI-generated and human-written abstracts in linguistics and computer science. Specifically, it examines and compares the readability and writing styles of these abstracts. This research addressed the critical need to distinguish between human-written and AI-generated abstracts, with significant implications for ethical standards, information security, and accountability in an increasingly AI-driven academic landscape.

Although prior research has compared AI-generated and human-written texts in fields like journalism, business, and technical communication [1], studies focusing specifically on academic abstracts remain limited. Moreover, few have examined AI-generated abstracts across disciplines with contrasting writing conventions, such as linguistics and computer science. This study addressed this gap by systematically comparing text readability and writing style differences between AI-generated and human-written abstracts, contributing to the broader discussion on AI’s role in academic writing. Specifically, this study aims to address the following research question: To what extent do human and AI-generated research article abstracts show similar and different readability and writing styles across disciplines?

## 2. Literature review

### 2.1 Research article abstract writing

Academic abstracts are vital for research dissemination, offering concise summaries that enable researchers to quickly assess study relevance [11,12]. Mastering abstract writing is a key academic skill, as abstracts serve as condensed representations of entire studies [13]. While general abstract patterns exist, disciplinary variations in structure may occur [14]. Typically encompassing the research problem, methodology, and key findings, abstract quality and structure can differ significantly across journals [15]. Enhancing abstract writing involves focusing on accuracy, appropriate length, and effective language use [12,16]. Suggestions for improvement include greater consistency and standardisation across journals, improved author and reviewer training, and increased emphasis on abstract quality [15]. Despite these challenges, a strong conceptual understanding of writing and structural framing may aid authors in effectively presenting their research [13].

### 2.2 Text readability and writing styles variations in AI-generated abstracts

Readability, the ease with which a text is understood, is often quantified using formulas such as the Flesch Reading Ease, Gunning Fog Index, and Coleman-Liau Index (Flesch, 1948).These metrics analyse sentence length, word complexity, and syllable count, making them applicable to academic texts [17]. Clear and readable abstracts can improve citation rates and knowledge transfer [18]. The increasing use of AI in text generation has driven interest in comparing the readability and writing style of AI-generated and human-written academic content [18–20]. While traditional readability formulas have limitations with academic writing’s specialized vocabulary and complex structures [21], they remain valuable tools for comparative analyses between human and AI-generated texts [22].

Writing style, encompassing lexical diversity and syntactic complexity, significantly impacts text quality and readability [23,24]. High lexical diversity can enrich a text, while appropriate syntactic complexity enhances clarity and precision. In academic writing, a well-crafted style is crucial for conveying complex ideas accessibly and authoritatively. Research on writing styles in academic contexts has examined linguistic complexity, finding that syntactic complexity, measured by sentence length and structure, and lexical complexity, referring to lexical diversity and sophistication, are key indicators of writing quality [25,26]. High-quality academic writing often exhibits greater syntactic complexity, lexical diversity, and the use of less frequent words [23,27].

### 2.3 Variations in human-written and AI-generated abstracts

Recent advancements in AI, particularly in large language models like GPT-4 and KIMI, have significantly improved automated academic text generation [28]. While AI-excels at grammatical accuracy and structural coherence [8], its output often follows a formulaic approach that lacks the nuances of human writing [29].

A key distinction lies in linguistic usage, whereby AI-generated texts tend to overuse nouns, pronouns, and adjectives, whereas human authors favour verbs and adverbs [7]. Furthermore, AI often employs complex vocabulary, while humans typically use simpler, more precise language [30]. These word choice differences contribute to variations in readability and stylistic fluidity.

AI-generated abstracts also tend to be less discipline-specific, struggling to incorporate field-specific nuances, which raises concerns for specialised academic writing [31]. Additionally, AI’s limited access to real-time data restricts the currency and relevance of the information it presents [29].

Comparative studies across disciplines reveal qualitative differences. Human-written abstracts generally offer more comprehensive discussions, emphasising context, methodology, and detailed results [31,32]. In contrast, AI-generated abstracts often prioritise clarity and purpose but lack depth. Interestingly, distinguishing between AI-generated and human-written abstracts can be challenging in some fields, like orthopaedic surgery [33,34]. Despite AI’s ability to produce scientifically accurate content, gaps in depth and overall quality persist, with AI-generated texts being more prone to redundancy and factual inaccuracies [35].

Disciplinary variations in writing styles further influence these comparisons. Fields such as Biology, Chemistry, Computer Science, and Psychology exhibit distinct trends in complexity and informativeness, with AI-generated texts often failing to match the depth and specificity of human work [36]. While AI continues to evolve as an academic writing tool, current limitations underscore the necessity of human oversight to ensure clarity, depth, and discipline-specific accuracy in academic abstracts.

### 2.4 Disciplinary variations in AI-generated abstracts

Disciplinary conventions significantly influence the effectiveness and readability of AI-generated abstracts. Different academic fields impose distinct expectations for writing style, rhetorical structure, and language use, shaping abstracts composition. While AI can mimic surface-level linguistic patterns, consistently adhering to discipline-specific norms remains challenging. Hard sciences, such as physics and engineering, often emphasise methodology and results, whereas soft sciences, including linguistics and sociology, prioritise theoretical discussions and argumentation [32]. These differences pose unique challenges for AI in generating discipline-appropriate abstracts.

In the social sciences, AI-generated abstracts exhibit linguistic limitations, including the overuse of uncommon academic vocabulary, limited subordinate structures, and a lack of syntactic diversity, resulting in formulaic and less natural text [37]. Humanities and social sciences tend to favour complex sentence structures and argument-driven writing, while science and engineering prioritise clarity and conciseness [38]. Interestingly, applied linguistics research has shown that experienced journal reviewers struggle to distinguish between AI-generated and human-written abstracts [10].

The increasing use of AI in academic writing also raises ethical concerns regarding authenticity, transparency, and potential misuse. While AI can aid in drafting and editing, its role in scientific communication requires careful management to maintain academic integrity [39]. Consequently, some publishers have implemented policies regulating AI use, from disclosure requirements to outright bans [40], reflecting growing concerns about responsible AI application in research dissemination.

## 3. Methods

### 3.1 Abstract data sources

This study analysed a corpus of 150 human-written and 150 Kimi-generated English academic abstracts. The principle of maximal variation was applied to guide the text selection among the top five journals of the respective field of study publications from the recent 3 years only. The human abstracts were sourced from ten Tier 1 journals indexed by WOS/SCI across two disciplines: linguistics and computer science, 5 from each discipline. The Kimi-generated abstracts were produced based on these corresponding articles after manually removing the original abstracts. Table 1 provides the information of the journals.

**Table 1:** Human-written and AI-generated corpus. 15 papers were selected from each of the 10 journals.

Discipline	Selected journal
Linguistics	Studies in Second Language Learning and Teaching
	Studies in Second Language Acquisition
	Applied Linguistics
	Language Learning
	Journal of Memory and Language
Computer Science	Journal of Big Data
	Information Systems Frontiers
	Data Science and Engineering
	International Journal of Systems Science
	Evolutionary Computation

Linguistics and computer science were selected due to their distinct academic writing and knowledge representation styles. Linguistics, a text-based discipline, emphasises qualitative analysis, narrative explanations, and theoretical discussions, typically employing a descriptive and discursive style focused on argumentation and detailed textual explanations [41]. Conversely, computer science, a symbolic-based field, often presented information through mathematical notation, algorithms, and formal structures, characterised by structured, concise, and formulaic writing incorporating technical terminology and symbolic representations [42,43].

### 3.2 Abstract generation

In this study, a total of 300 abstracts from 10 high-impact journals (linguistics and computer science) were selected as a control corpus of well-written abstracts.

The full-text article (PDF) was uploaded after manually removing the original abstract, so the model received only the main body of the paper and had no access to the human-written abstract. The AI tool Kimi (version 2.0) then generated 150 abstracts based on the uploaded file, using the prompt: “Please write an abstract of no more than 300 words based on the attached article.” All AI-generated abstracts, along with the corresponding human-written abstracts, were analysed using readability scoring systems and writing style evaluation tools. This allowed for a direct comparison of text readability and writing style scores between the two sources across the two disciplines.

### 3.3 Abstract evaluation

#### 3.3.1 Automated machine assessment.

AI-generated and human-written abstracts were compared in terms of text readability and writing styles. Each abstract was evaluated using an online computational tool, the Readability Scoring System (https://readabilityformulas.com/readability-scoring-system.php) developed by Brain Scott (2024).

In this scoring system, text readability was further assessed through nine different readability metrics, namely, Automated Readability Index (ARI), Flesch Reading Ease, Gunning Fog Index, Flesch-Kincaid Grade Level, Coleman-Liau Index, SMOG Index, Linsear Write Readability Formula, FORCAST Readability Formula, Average Reading Level Consensus Calc.

Writing style was analysed through lexical diversity and syntactic complexity. The details are presented in Table 2.

**Table 2:** Measures of text readability and writing styles.

Abstract evaluation	Measures
Text readability assessment	Automated Readability Index (ARI) Flesch Reading Ease Gunning Fog Index Flesch-Kincaid Grade Level Coleman-Liau Index SMOG Index Linsear Write Readability Formula FORCAST Readability Formula Average Reading Level Consensus Calc
Write Style Analysis	Lexical Diversity Diversity Analysis

Readability Scoring System analysed English-language text and scored the “reading ease” or “reading difficulty” of the text based on popular readability formulas.

### Text readability assessment

- **Automated Readability Index (ARI):** Determines the grade level required to comprehend a text by analysing sentence length and character count.
- **Flesch Reading Ease:** Assigns a readability score between 0 and 100, where higher values indicate easier text and lower values suggest greater difficulty.
- **Gunning Fog Index:** Estimates the grade level based on sentence length and the percentage of complex words (words with three or more syllables).
- **Flesch-Kincaid Grade Level:** Similar to the Flesch Reading Ease formula but provides a U.S. school grade level for readability.
- **Coleman-Liau Index:** Evaluates readability based on the number of characters per word and words per sentence, without relying on syllable counting.
- **SMOG Index (Simple Measure of Gobbledygook):** Estimates the reading level required to understand a text by calculating the number of polysyllabic words.
- **Linsear Write Formula:** Assesses readability based on sentence structure and word complexity, often used for technical and instructional writing.
- **FORCAST Readability Formula:** Designed for specialised or technical texts, it predicts comprehension difficulty based on the use of simple and complex words.
- **Average Readability Consensus Calculation:** Aggregates multiple readability scores to provide an overall reading level estimate.

### Writing style analysis

To assess writing styles, this study examines lexical density and diversity, which provide insights into vocabulary richness, complexity, and formality:

- **Lexical Density:** Measures the proportion of content words (nouns, adjectives, verbs, and adverbs) relative to the total number of words. Higher lexical density indicates more information-rich, formal writing, while lower density suggests a conversational style.
- **Lexical Diversity:** Evaluates the variety of words used in a text by calculating the ratio of unique words to total words. A higher lexical diversity score signifies a broader vocabulary range, whereas a lower score may indicate repetitive language or domain-specific jargon.

#### 3.3.2 Experts evaluation.

In addition to quantitative analyses, a qualitative evaluation was conducted using expert evaluation. Three experts experienced in scientific writing and publishing reviewed AI-generated and human-written abstracts, focusing on structural readability (sentence length, clause embedding, word length, and information packing) and lexicon-stylistic features (lexical variation, balance between repetition and variation, and functional use of vocabulary) (see Table 3). The evaluation aimed to identify how abstracts realized complex constructs, multi-variable relationships, and contextual information within each discipline. Experts’ judgments highlighted discipline-specific patterns in abstract writing and provided insights that complemented quantitative measures of syntactic complexity and lexical diversity.

**Table 3:** Pre-defined themes and sub-themes used in experts evaluation.

Themes	Sub-theme
Text readability	Sentence length and clause embedding
	Word length and multisyllabic words
	Information packing within sentences
Writing features	Lexical variation
	Balance between repetition and variation
	Functional use of lexical diversity

### 3.4 Statistics and visualisation

Data generated were analysed using SPSS 27. Given the non-normal distribution of data (Shapiro-Wilk, p < 0.05), non-parametric statistical methods were employed. Since each AI-generated abstract was paired with its human counterpart from the same article (forming dependent observations), the Wilcoxon signed-rank test was used to compare the two related groups. Statistical significance was reported using median values (consistent with non-parametric testing conventions) and p-values to highlight key differences.

To effectively present the disparities in the data, violin plots were generated using Jamovi 2.6.23., providing a visual representation of the distribution, central tendency, and variability of the data across different sources.

### 3.5 Ethical statement

The study does not involve animal or human participants.

## 4. Results

The results were organised into three sections, aligned with the study’s objectives. The first section presented a comparison of readability and writing styles between human-written and AI-generated abstracts. The second section focused on intra-disciplinary comparisons within linguistics and computer science. The third section extended the analysis to an interdisciplinary perspective, comparing AI-generated abstracts across the two fields.

### 4.1 Variations between human-written abstracts and AI-generated abstracts

**Fig 1:**
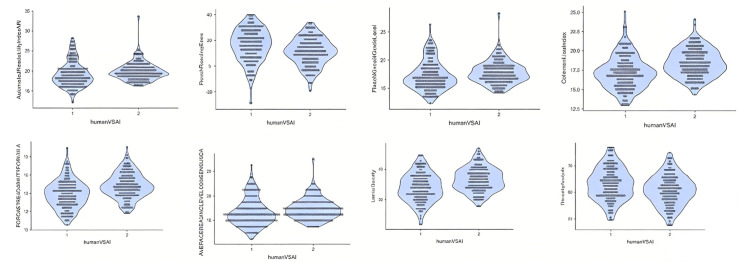
Violin plot of Human-written and AI-generated Abstract Comparison. (1) Automated Readability Index (2) Flesch Reading Ease; (3) Flesch-Kincaid Grade Level; (4) Coleman-Liau Index; (5) FORCAST Readability Formula; (6) Average Reading Level Consensus Calc; (7) Lexical Density; (8) Diversity Analysis.

To compare human-written abstracts and AI-generated abstracts, 300 texts were used as corpus data. The results (median values and p value) are illustrated in Table 3 and visualised in Fig 1.

Table 4 presented the percentiles 50^th^ median value of readability and writing style measures for human-written and AI-generated abstracts, along with p-values indicating statistically significant differences in 8 indices versus similarities in 3 indices. AI-generated abstracts scored significantly higher on the Automated Readability Index (ARI), Flesch-Kincaid Grade Level, Coleman-Liau Index, and Readability Formula (*p* < 0.05), indicating greater complexity in sentence structure and vocabulary. For instance, the Coleman-Liau Index (Human 17.01 vs. AI 18.41, *p* < 0.001) and FORCAST Readability Formula (Human 12.93 vs. AI 13.36, *p* < 0.001) suggest AI uses longer words and sentences, reducing readability. Additionally, the lower Flesch Reading Ease score for AI (Human 17.00 vs. AI 11.50, *p* < 0.001) further confirms this difficulty, likely due to longer sentence structures and multi-syllabic words.

**Table 4:** Readability and writing style measures across the human and AI-generated abstracts.

measure	Human-written	AI-generated	*P* value
Automated Readability Index (ARI)	18.60	19.63	**.013**
Flesch Reading Ease	17.00	11.50	**<.001**
Gunning Fog Index	20.20	20.80	.306
Flesch-Kincaid Grade Level	16.82	17.62	**.029**
Coleman-Liau Index	17.01	18.41	**<.001**
SMOG Index	14.72	15.23	.183
Linsear Write Readability Formula	18.80	19.00	.870
FORCAST Readability Formula	12.93	13.36	**<.001**
Average Reading Level Consensus Calc	17.00	18.00	**.008**
Lexical Density	63.70%	66.30%	**<.001**
Diversity Analysis	63.70%	60.95%	**<.001**

a. The significance level is.050.

In terms of writing style, AI-generated abstracts had significantly higher lexical density (Human 63.70% vs. AI 66.30%, *p* < 0.001), suggesting a greater proportion of unique words and a more academic tone. However, their lower lexical diversity was significantly lower (Human 63.70% vs. AI 60.95%, *p* < 0.001), indicating more word repetition, possibly due to AI’s reliance on structured templates. While AI exhibited higher lexical density, its lower lexical diversity suggests a more rigid and repetitive writing style. These differences suggested AI-generated abstracts align more with formal academic writing in structure and vocabulary but may lack the natural variation and readability of human-written abstracts. The violin plot in Fig 1 further revealed the disparities in text readability and writing styles in the eight indices.

Expert evaluations aligned with the quantitative analyses, confirming clear differences between human-written and AI-generated abstracts. Experts noted that human-written abstracts employ complex sentence structures with embedded clauses and discipline-specific terminology, enabling nuanced presentation of background, methodology, and findings. This corresponds with the higher syntactic complexity and lexical diversity observed in the quantitative metrics. In contrast, AI-generated abstracts use shorter, simpler sentences and basic, repetitive vocabulary, enhancing readability but reducing informational depth and precision.

*It involved the* ***participation of 121 Chinese students, who are English language learners****, at a university in the United States.* ***Haws et al.’s (2010) questionnaire was used to examine the participants’ regulatory dispositions,*** *and* ***a judgment task was adapted from Bardovi-Harlig and Dörnyei (1998)*** *to assess participants’ awareness of grammatical and pragmatic errors, as well as the severity of each type of error. (Paper 2-human-written)*

*The study involved* ***121 ESL speakers*** *at a U.S. university, using* ***a regulatory focus questionnaire****, an* ***error judgment task****, and a demographic questionnaire.(Paper 2-AI-generated)*

Experts highlighted that the functional lexical variation in human-written abstracts allows distinct research elements to be clearly conveyed, whereas AI-generated summaries, though concise, provide less differentiation between research components. Overall, expert judgment supports the quantitative findings: human-written abstracts prioritize scholarly depth and precision, while AI-generated abstracts favor accessibility and rapid comprehension.

*Given the lack of research into native-speakerism among teachers of languages other than English (LOTEs), this qualitative study aims to* ***bridge the gap*** *by* ***investigating the discriminatory and inclusive language*** *employed in* ***online recruitment*** *for* ***post-secondary institution instructors*** *of LOTEs. (Paper 6-human-written)*

*This study examines* ***the prevalence*** *of native-speakerism in* ***job advertisements*** *for language teachers in the United States, focusing on languages other than English (LOTEs). (Paper 6-AI-generated)*

Consequently, it can be concluded that AI-generated abstracts scored higher on multiple readability indices, indicating they are generally more complex and harder to read than human-written abstracts.

### 4.2 Discipline-specific variations in text readability and writing styles

**Fig 2:**
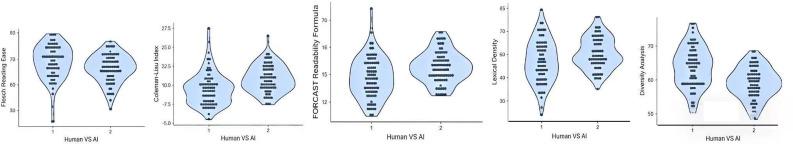
Violin-plot of human-written and AI-generated in Linguistics. (1) Flesch Reading Ease; (2) Coleman-Liau Index; (3) FORCAST Readability Formula; (4) Lexical Density; (5) Diversity Analysis.

**Fig 3:**
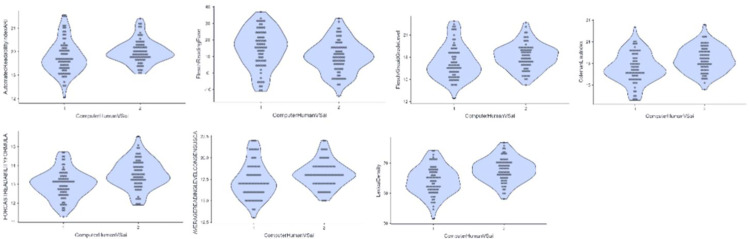
Violin-plot of human-written and AI-generated in Computer Science. (1) Automated Readability Index; (2) Flesch Reading Ease; (3) Flesch-Kincaid Grade Level; (4) Coleman-Liau; (5) FORCAST Readability Formula;(6) Average Reading Level Consensus Calc; (7) Lexical Density.

This study compared AI and human performance within linguistics and computer science via Wilcoxon signed-rank test with descriptive statistics (percentiles 50^th^ median value) and P value. The results were presented in Tables 4 and 5 and visualised in Figs 2 and 3.

**Table 5:** Variations of text readability and writing styles in linguistic journals.

Measure	Human-written	AI-generated	*P* value
Automated Readability Index (ARI)	18.40	19.42	.246
Flesch Reading Ease	20.00	14.00	**<.001**
Gunning Fog Index	19.90	20.10	.910
Flesch-Kincaid Grade Level	16.77	17.13	.336
Coleman-Liau Index	17.00	18.38	**<.001**
SMOG Index	14.49	14.87	.720
Linsear Write Readability Formula	19.10	18.74	.381
FORCAST Readability Formula	12.88	13.31	**<.001**
Average Reading Level Consensus Calc	17.00	17.00	.214
Lexical Density	63.50%	64.60%	**.001**
Diversity Analysis	64.70%	59.40%	**<.001**

a. The significance level is.050.

Table 5 revealed significant differences in five indices and similar readability and writing styles in six indices between human-written and AI-generated abstracts in linguistics. These five differing indices mirrored the overall findings in Section 4.1, while the other three significant indices from that section were not significantly within linguistics alone.

Consistent with Section 4.1, AI-generated linguistics abstracts had significantly lower Flesch Reading Ease scores (*p* < 0.05), indicating lower readability. Additionally, the Coleman-Liau Index was significantly higher for AI-generated abstracts (*p* < 0.001), suggesting more long words and complex sentences. The Forecast Readability Formula also showed significantly higher complexity for AI (*p* < 0.001). Regarding writing style, AI-generated abstracts had significantly higher lexical density (*p* < 0.05), indicating more content words. However, lexical diversity was significantly lower for AI-generated abstracts (*p* < 0.001), suggesting more repetitive word use. Fig 2 visually highlights these five differences. These findings suggest that while AI-generated linguistics abstracts can match human complexity in some ways, they tend to be less readable and less lexically diverse [44].

The results indicated that AI-generated abstracts in linguistics can closely mimic the human complexity in certain aspects but are generally less readable and employ less varied language. The higher complexity and lower readability, as indicated by the Coleman-Liau Index and FORCAST Readability Formula, may stem from AI’s tendency to use more complex sentence structures and vocabulary, potentially increasing information density but reducing accessibility [45]. The higher lexical density suggests a greater concentration of content words, which could be beneficial for conveying detailed information but challenging for readers with varying expertise. The significantly lower lexical diversity in AI abstracts suggests that human-written abstracts employ a broader range of vocabulary and sentence structures, potentially improving reader engagement and comprehension, a critical aspect of academic writing that AI currently struggles to fully replicate.

In comparison with linguistics shown in Table 4, Table 5 shows the results of variations of text readability and writing style in computer science journals.

Table 6 shows that only four metrics (Gunning Fog Index, SMOG Index, Linsear Write Readability Formula and Diversity analysis) did not significantly differ between human-written and AI-generated abstracts in computer science. Conversely, seven metrics showed significant differences.

**Table 6:** Variations of text readability and writing styles in computer science journals.

Measure	Human-written	AI-generated	*P* value
Automated Readability Index (ARI)	18.61	19.67	**.016**
Flesch Reading Ease	16.00	10.00	**.002**
Gunning Fog Index	20.30	21.30	.181
Flesch-Kincaid Grade Level	16.99	18.13	**.032**
Coleman-Liau Index	17.02	18.46	**<.001**
SMOG Index	14.87	15.45	.117
Linsear Write Readability Formula	18.72	19.09	.518
Readability Formula	13.09	13.49	**<.001**
Average Reading Level Consensus Calc	17.00	18.00	**.011**
Lexical Density	64.20%	67.80%	**<.001**
Diversity Analysis	62.70%	62.70%	.931

a. The significance level is.050.

Table 6 and Fig 3 indicate that AI-generated abstracts generally had higher readability scores on metrics such as the Automated Readability Index (AI: 19.67 vs. Human: 18.61, *p* < 0.01) and Flesch-Kincaid Grade Level (AI: 18.13 vs. Human: 16:99, *p* < .05). Additionally, AI showed higher lexical density (AI:67.80% vs. Human: 64.20%, *p* < 0.01), suggesting a greater use of content words. However, the Flesch Reading Ease score was lower for AI (10.00 vs. Human:16.00, *p < 0.005*), indicating lower readability.

In terms of writing style, AI-generated computer science abstracts tend to prioritise clarity and purpose but often lack depth. They scored higher on the Coleman-Liau Index (AI:18.46 vs. Human: 17.02) and SMOG Index (AI:15.45 vs. Human: 14.87, *p* < 0.05), further suggesting complexity. Despite these higher readability scores, AI abstracts often miss the depth and specificity of human-written ones, which tend to incorporate more verbs and adverbs, enhancing the narrative flow and coherence of the text.

Experts observed that human-written abstracts typically integrate multiple analytical dimensions within single, syntactically complex sentences, allowing abstract constructs, methodological design, and variable distinctions to be foregrounded simultaneously (e.g., embedding participant scope, data volume, and measurement levels in one sentence). This pattern aligns with the higher syntactic complexity and lexical diversity found in the quantitative results.

By contrast, AI-generated linguistics abstracts tend to distribute the same information across shorter, sequential sentences. Although this restructuring improves readability, experts noted that it reduces information density and weakens the functional differentiation of research components, resulting in a flatter representation of theoretical and methodological relationships. It is indicated that human-written linguistics abstracts emphasize disciplinary depth and analytical precision, whereas AI-generated abstracts prioritize accessibility.

*Across two weeks, 16 Hong Kong EFL students completed* ***pre-and post-trait-level surveys*** *and generated 1,120* ***state-level responses*** *via the experience sampling method (ESM). (Paper 8-human-written)*

*Using an experience sampling method (ESM), the research collected real-time data from 16 Hong Kong EFL students over 14 days to examine* ***both trait (in-class, out-of-class, and digital)*** *and* ***state (momentary) levels*** *of L2 WTC. (Paper 8-AI-generated)*

Expert evaluations also revealed systematic differences within computer science journals. Human-written abstracts use long, information-dense sentences that integrate system types, control strategies, theoretical properties, and experimental results, reflecting the field’s emphasis on precise modeling and algorithmic specification. AI-generated abstracts split the same information into shorter sentences with more general technical vocabulary, enhancing readability but weakening differentiation among methods, system characteristics, and outcomes. Overall, human-written CS abstracts prioritize technical precision and integrated presentation, whereas AI-generated abstracts favor clarity and linear readability.

*In order to guarantee the stability of the closed-loop system, a generalised nonlinear proportional differential controller is designed to configure the system into a desired linear constant system.* ***Then, an explicit reference governor for high-order system is introduced*** *to modify the reference signal such that the system state and the state derivatives of certain orders always remain within a prescribed constraint set. (Paper 134-human-written)*

*The control strategy* ***involves two steps****: first, designing a generalized nonlinear proportional differential (PD) controller to stabilize the system; second, constructing an ERG to modify the reference signal to ensure state constraints are not violated. (Paper 134-AI-generated)*

In conclusion, while AI can replicate certain aspects of human writing in computer science, significant differences exist across eight metrics, highlighting AI’s limitations in mimicking human writing styles in this field. AI-generated abstracts are generally more complex and less readable, suggesting that AI struggles to capture the nuances of human writing in computer science. This aligns with previous research that found AI often prioritized clarity over depth. For instance, a comparative genre analysis of AI-generated and scholar-written abstracts for English review articles in international journals found that AI-generated texts often prioritise clarity and purpose statements but may lack the depth and critical nuance found in human-written texts [32]. This is particularly relevant in specialised fields such as computer science, where the ability to convey complex ideas with precision and clarity is crucial. The higher readability scores and lexical density in AI-generated abstracts suggest an aptitude for formal, structured content. However, the lower Flesch Reading Ease scores indicate potential readability issues due to complex sentences and vocabulary, mirroring findings in other fields like orthopaedic surgery [33].

### 4.3 Text readability and writing styles variations between two disciplines

**Fig 4:**
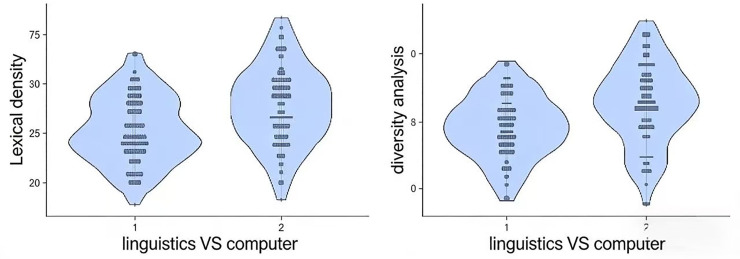
Violin-plot of AI-generated abstracts across two disciplines. (1) Lexical Density; (2) Diversity Analysis.

Interdisciplinary analysis was conducted across two disciplines to identify the similarities and differences of text readability and writing styles of AI-generated abstracts. The results are illustrated in Table 7 and Fig 4.

**Table 7:** AI-generated abstract comparison in linguistics and computer science.

Measure	Linguistics	Computer science	*P* value
Automated Readability Index (ARI)	19.42	19.67	.714
Flesch Reading Ease	14.00	10.00	.131
Gunning Fog Index	20.10	21.30	.060
Flesch-Kincaid Grade Level	17.13	18.13	.121
Coleman-Liau Index	18.38	18.46	.810
SMOG Index	14.87	15.45	.080
Linsear Write Readability Formula	18.74	19.09	.182
FORCAST Readability Formula	13.31	13.49	.065
Average Reading Level Consensus Calc	17.00	18.00	.167
Lexical Density	64.60%	67.80%	**<.001**
Diversity Analysis	59.40%	62.70%	**<.001**

Table 7 compares AI-generated abstracts in linguistics and computer science across various readability and lexical measures. The results showed significant differences in 2 7 out of 11 indices (*p* < 0.001) when comparing AI-generated abstracts across linguistics and computer science. AI-generated abstracts in computer science and linguistics exhibit overall similarity across most readability metrics, as indicated by non-significant differences in measures such as the Automated Readability Index (19.42 vs. 19.67, p = .714), Flesch Reading Ease (14.00 vs. 10.00, p = .131), and Flesch-Kincaid Grade Level (17.13 vs. 18.13, p = .121), among others. However, notable distinctions emerge in lexical characteristics: computer science abstracts demonstrate significantly higher lexical density (67.80% vs. 64.60%, p < .001) and lexical diversity (62.70% vs. 59.40%, p < .001) compared to those in linguistics. Thus, while the two fields’ AI-generated abstracts are comparable in readability, they differ markedly in lexical richness.

Expert evaluation of AI-generated abstracts across disciplines revealed differences in readability and writing style between linguistics and computer science journals, which align with the findings from the quantitative analyses. In linguistics journals, AI abstracts use short, sequential sentences and general academic vocabulary, which improves readability but reduces differentiation of theoretical constructs, methodological details, and contextual factors. In computer science, AI abstracts also employ concise sentences but focus on stepwise procedural description and technical operations, enhancing procedural clarity while limiting conceptual nuance. AI-generated abstracts prioritize readability across disciplines, but linguistics emphasizes construct and context clarity, whereas computer science emphasizes methods and system specification, reflecting discipline-specific writing conventions.

*This study investigates the impact of implicit corrective feedback, specifically recasts, on the production of lexical stress in L2 English among Arabic-speaking learners. (Paper 9-AI-generated)*

*This study aims to optimize and validate a multi-parametric wearable platform for stress level assessment using physiological signals. (Paper 93-AI-generated)*

In conclusion, AI-generated computer science abstracts were significantly more complex and less readable than those in linguistics, as reflected in higher Gunning Fog Index and the Flesch-Kincaid Grade Level scores. They also exhibited higher lexical density and greater word repetition compared to linguistics abstracts. This finding aligned with previous research highlighting ChatGPT’s limitations in academic writing. Specifically, Tudino and Qin (2024) identified issues such as the overuse of infrequent “academic” vocabulary, limited use of subordination, and a lack of syntactic and semantic diversity. While higher lexical density may suggest richer vocabulary, it can also reduce readability. In contrast, AI-generated linguistics abstracts, though still complex, tended to be more readable and less dense, possibly due to the nature of the field.

## 5. Discussion

Despite the rapid adoption of AI tools in academic writing, existing research has yet to systematically compare the readability and stylistic features of AI-generated versus human-written academic abstracts—particularly across distinct disciplines. This study addresses this gap by analysing AI-generated and human-written abstracts in linguistics and computer science, with core findings showing that AI-generated abstracts exhibit significantly lower readability across eight key metrics and display discipline-specific variations in readability patterns, while struggling with lexical diversity relative to human-written counterparts.

### 5.1 Human-written and AI-generated abstracts variations

The present study revealed that AI-generated research abstracts were significantly different from human-written ones in 6 readability metrics (including the Automated Readability Index, Flesch Reading Ease, and Lexical Diversity Analysis) and 2 writing style indices (lexical density and lexical diversity). AI tended to use longer sentences and more complex structures, reducing the readability, making them difficult to read and comprehend. The narrower use of these features in GenAI writing may result from the models’ training data and algorithms [6]. Additionally, AI-generated abstracts displayed higher level of lexical density and lower lexical diversity, suggesting a more rigid and repetitive writing style.

Our results with KIMI 2.0 are consistent with Huang’s (2025) findings from ChatGPT-4o and OpenAI-o1: both models tend to prioritize syntactic complexity over communicative clarity, as they learn to mimic formal linguistic registers from training data but lack an inherent grasp of reader needs [46]. This tendency is particularly pronounced in academic contexts, where AI may over-utilize technical jargon or convoluted sentence structures to signal “scholarly tone,” as observed in comparative studies of AI and human research papers.

The reduced lexical diversity in KIMI 2.0-generated abstracts further resonate with findings by Mo (2025), who demonstrated that ChatGPT, a representative large language model, tends to generate text with narrower vocabulary ranges compared to human writers, relying on high-frequency terms to maintain fluency. In academic abstracts—where conciseness and precision are paramount—this limitation risks obscuring nuanced contributions or alienating readers unfamiliar with field-specific terminology. These results reinforce calls for “readability-aware” AI training, such as integrating metrics like the Flesch-Kincaid Grade Level into loss functions to balance technical accuracy with accessibility.

### 5.2 Intra-disciplinary variations

In line with the findings in 5.1, the comparisons between AI and human performance within linguistics and computer science indicated that AI-generated linguistics abstracts had significantly lower Flesch Reading Ease scores (*p* < 0.05), indicating lower readability. And lexical diversity was significantly lower for AI-generated abstracts (*p* < 0.001), suggesting more repetitive word use.

Linguistics, a field centered on language structure and meaning, showed discrepancies in metrics like Lexical Density, which aligns with findings by Mindner, et al. (2023) that ChatGPT struggles to replicate the nuanced lexical choices critical to linguistic analysis, often over-simplifying theoretical distinctions or overusing jargon [19]. In contrast, computer science—characterized by algorithmic descriptions and technical rigor—exhibited broader divergences, including in the SMOG Index, which measures syllable complexity. This echoes observations by Chen et al. (2024) that AI-generated technical prose in computer science tends to over-embed complex terms (e.g., “neural network architectures,” “computational complexity”) without contextual scaffolding, inflating readability scores beyond typical human-written norms [47].

These variations underscore the need for domain-adapted AI training, as Xie et al. (2023) argue that models fine-tuned on discipline-specific corpora better align with field-specific readability norms. The lower overall readability of AI-generated abstracts—evidenced by higher Flesch-Kincaid Grade Levels and lower Flesch Reading Ease scores—also supports Benber et al.’s (2024) observation that LLMs AI prioritize syntactic complexity over communicative clarity, a tendency that is particularly problematic in academic contexts where accessibility is key to knowledge dissemination.

Notably, both fields showed significant differences in the FORCAST Readability Formula, a metric designed for technical texts. This suggests that while AI may grasp discipline-specific terminology, it fails to calibrate the “density” of such terms to match human conventions—likely because training data includes uneven distributions of disciplinary texts, leaving gaps in AI’s ability to emulate field-specific stylistic norms.

### 5.3 Interdisciplinary variations

Interdisciplinary analysis indicated non-significant differences across nine readability metrics support the notion that AI has internalized general features of academic discourse, such as structural conventions and syntactic patterns. This finding aligns with research by Chen et al. (2024), who argued that that ChatGPT performs equally well as human participants in four out of the five tested pragmalinguistic features and five out of six sociopragmatic features. Additionally, the conversations generated by ChatGPT exhibit higher syntactic diversity and a greater sense of formality compared to those written by humans [47].

However, the persistent deficit in lexical diversity across disciplines echoes critiques by Du, et al. (2025), who highlighted that AI’s reliance on statistical co-occurrence patterns leads to repetitive phrasing, undermining the rhetorical function of abstracts—to engage readers through varied and precise language. The increased rates of repetition seen in texts produced by AI point to a potential constraint in their capacity to build a diverse and contextually relevant lexicon [48]. This limitation is particularly striking when compared to human abstracts, which consistently demonstrate higher lexical variation to emphasize novelty.

Addressing this gap may require hybrid approaches: combining AI’s efficiency in drafting with human oversight to refine lexical choices, as proposed by Hemmer, et al. (2023), who found that such collaboration improved both readability and diversity in academic writing [49,50]. Additionally, fine-tuning models on discipline-specific corpora annotated for lexical variation could help AI better emulate the nuanced word choice that distinguishes effective academic abstracts.

## 6. Conclusion

This study compared the readability and writing styles of human-written and AI-generated research abstracts in linguistics and computer science. AI-generated abstracts were found to be more complex and less readable across multiple metrics. Intra-disciplinary analysis showed AI struggled to replicate human writing in computer science more than in linguistics, while inter-disciplinary comparison revealed broader challenges in achieving coherence and natural variation. These findings underscored current limitations of AI in academic writing, particularly in technical fields requiring precise and accessible communication. Although AI tools offer potential benefits in assisting with abstract generation, they require further refinement to better align with human writing conventions.

## 7. Limitations and recommendations for future directions

This study has several limitations. First, it only used Kimi for AI-generated abstracts, without comparing it to other models such as ChatGPT or DeepSeek. Future research should explore differences across multiple AI systems for a more comprehensive understanding of readability and writing style variations. Second, the study focused on only two disciplines, limiting the generalisability of the findings.

Given these limitations, several key recommendations emerge. First, future researchers could explore strategies to improve AI-generated academic texts by fine-tuning language models for enhanced readability and stylistic adaptability across disciplines. Second, researchers could broaden the analysis to include more technical fields such as physics and engineering, comparing them with social sciences to examine discipline-specific trends in AI-generated and human-written abstracts. Third, academic institutions and educators may integrate AI into research workflows by providing targeted training that teaches researchers to critically evaluate AI outputs, establishing mentorship programs for ethical AI use, and investing in discipline-specific AI tools. This integration should not only be widespread but also deep, ensuring that all facets of academic work are touched by AI’s transformative capabilities. Lastly, journal editors should set clear rules for authors to disclose AI use, train reviewers to assess AI-generated content, and collaborate with developers to align AI tools with journal standards.

## Supporting information

S1 File Data. (XLSX)
